# Successful Application of an Insole with a Metatarsal Inhibition Bar and Deep Heel Cup for Improving Gait Dysfunction in a Patient with Poor Coordination with Disrupted Corticoreticular Tracts: A Case Report

**DOI:** 10.3390/children8050320

**Published:** 2021-04-22

**Authors:** Su Min Son, Jung Won Lee, Min Cheol Chang

**Affiliations:** Department of Rehabilitation Medicine, College of Medicine, Yeungnam University, Daegu 42415, Korea; sumin430@hanmail.net (S.M.S.); jwlee22c@naver.com (J.W.L.)

**Keywords:** coordination, insole, metatarsal inhibition bar, gait dysfunction, diffusion tensor tractography, corticoreticulospinal tract

## Abstract

We report the successful management of gait dysfunction in a patient with coordination problems using an insole with a metatarsal inhibition bar (MIB) and a deep heel cup. Furthermore, we investigated the state of the neural tracts via diffusion tensor tractography (DTT). A 23-month-old boy with gait dysfunction presented with toe walking with a wide base and decreased hip flexion. Motor weakness or spasticity was not observed. Conventional brain magnetic resonance imaging did not reveal any abnormal findings, but DTT revealed disrupted bilateral corticoreticulospinal tracts (CRTs). No abnormalities were observed in the corticospinal tract or the medial lemniscus. We applied a custom-made insole with an MIB and a deep heel cup. Immediately after application, the patient’s gait pattern stabilized significantly and was nearly normalized. Our therapeutic experience indicates that the application of an insole with an MIB and deep heel cups could be beneficial for patients with coordination problems and gait dysfunction. Our DTT results showed that CRTs could be the causative brain pathology for gait dysfunction in patients with coordination problems.

## 1. Introduction

Coordination is very important for development and can affect many aspects. If children show problems, clinicians can consider the occurrence of developmental coordination disorder (DCD). This is a neurodevelopmental condition characterized by problems in performing coordinated motor skills, such as walking or crawling, which may be delayed in the early developmental period [1,2]. Coordination difficulties can affect gross or fine motor movements, or both. In addition, motor skill deficits can interfere with executive functions in activities of daily living and academic achievement [2]. Children with poor coordination can experience various types of difficulty associated with motor performance, such as postural control, dressing, scissoring, tying shoelaces, and other self-care activities. DCD occurs in approximately 5%–6% of school-aged children [3,4], and it is usually diagnosed in pediatric patients aged >5 years.

Although some studies have reported neural impairments in the immature brain of pediatric patients with coordination problems, the pathogenesis related to them or DCD has not been clearly demonstrated [5,6,7,8]. Many patients with DCD usually have no definite abnormal lesions on conventional brain magnetic resonance imaging (MRI) [2,3]. However, diffusion tensor imaging (DTI) can evaluate the state of the neural tract related to functional problems that cannot be explained using conventional MRI, even in the immature brain [9,10].

In this study, we used diffusion tensor tractography (DTT) to evaluate the causative brain pathology for poor coordination, including gait dysfunction, and investigated the state of neural tracts, such as the corticospinal tract (CST), corticoreticulospinal tract (CRT), and medial lemniscus (ML), which are known to be related to major motor functions [10]. We also reported the successful application of an insole with a metatarsal inhibition bar (MIB) and a deep heel cup to improve gait stability.

## 2. Case Presentation

A 23-month-old boy was brought to the Rehabilitation Medicine Department of Yeungnam University Hospital for the treatment of gait dysfunction. He could walk independently, but his gait pattern was unstable. During gait, toe walking (decreased ankle dorsiflexion), decreased hip flexion, and a wide base were noted (Supplementary Video S1). Physical examination revealed no definite motor weakness or spasticity. Deep tendon reflexes, including those of the triceps, biceps, ankle, and knee jerks, were normal and had no pathological reflexes. He was born at 39 weeks of gestation, and had no specific perinatal history or medical diagnosis, such as epilepsy. However, his mother reported that he showed a delay in developmental milestones, except for head control. His bimanual coordination abilities decreased remarkably. He often dropped objects from his hands. He could not use a spoon or fork well while eating. His mother reported that she tried to teach him to use a spoon, but he did not learn well; thus, his mother fed him. Motor skill deficits were more prominent than intellectual disabilities. Conventional brain and spinal MRI performed under natural sleep revealed no abnormal findings. Genetic studies and blood tests showed no evidence of muscular dystrophy. The proprioception test failed because of poor patient cooperation. We could not perform electrodiagnostic testing (electromyography and nerve conduction study) because of the refusal of the patient’s parents.

**Figure 1:**
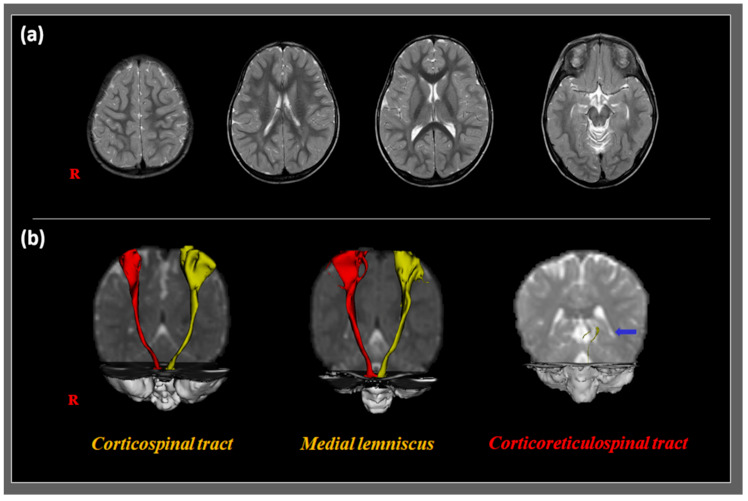
T2-weighted magnetic resonance images (upper row) and diffusion tensor tractography (DTT) images (lower row) of a 23-month-old boy with developmental coordination disorder showing gait dysfunction. (**a**) T2-weighted magnetic resonance images show no abnormal lesions. (**b**) DTT images revealing a disrupted corticoreticulospinal tract (blue arrow). However, the corticospinal tract and medial lemniscus show preserved integrity.

Since our patient was under five years of age, he could not be diagnosed with DCD. However, various clinical features showed a remarkable coordination problem, similar to that of DCD. To analyze the causes of gait dysfunction and poor coordination in the patient, we evaluated the state of the neural tracts using DTI. We obtained DTI data on a 1.5-T scanner (Gyroscan Intera; Philips Medical Systems, Best, Netherlands) with a synergy-L Sensitivity Encoding 6-channel head coil (Roche, Basel, Switzerland). We acquired 67 axial slices, which were parallel to the anteroposterior commissure line (matrix = 128 × 128; thickness = 2.3 mm; field of view = 221 × 221 mm^2^; repetition time = 10,726 ms; echo time = 76 ms; echo-planar imaging factor = 67; b = 1000 s/mm^2^; number of excitations = 1). We used the Oxford Center for Functional MRI of the Brain Software Library for the analysis of DTI data (free software in fmrib. ox. ac. uk/fsl). Based on a multi-fiber model, fiber tracking was conducted using a probabilistic tractography method. In addition, we applied the following parameters: streamline samples, 5000; length of step, 0.5 mm; thresholds of curvature, 0.2. The CST, CRT, and ML were depicted by the selection of fibers passing through the target and seed regions of interest (ROIs) [11,12]. To evaluate the CST state, we located a seed ROI of the CST on the portion of the level of the anterior mid-pons, in which the CST passed on a two-dimensional color map. The target ROI was placed on the portion of the anterior lower pons through which the CST passed. To investigate the integrity of the CRT, we placed a seed ROI on the reticular formation. The first and second target ROIs were located on the midbrain tegmentum and premotor cortex in Brodmann area 6, respectively. To investigate the state of the ML, the seed and target ROIs were located in the medial posterior region of the medullary pyramids and the ventral posterolateral nucleus of the thalamus, respectively. Fiber tracking of the CST, CRT, and ML was initially conducted at the center of the seed voxel. For the analysis, we set a fractional anisotropy (FA) of >0.2, ending at the voxel with a fiber assignment of <0.2. In addition, we set a tract-turning ankle with <60°. The CST and ML appeared to have a preserved state in the cortex and did not demonstrate any abnormal findings on either side. However, the CRTs appeared to be disrupted in both the hemispheres (Figure 1).

**Figure 2:**
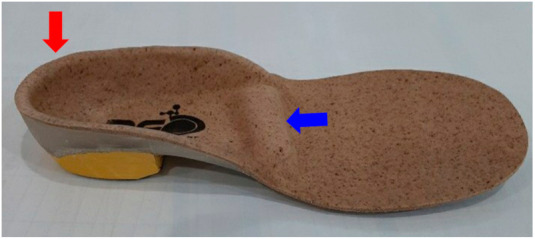
Insole with a metatarsal inhibition bar (blue arrow) and a deep heel cup (red arrow).

To improve gait function, we applied a rigid custom-made insole with an MIB and a deep heel cup bilaterally to the feet (Figure 2) to reduce weight-bearing and toe-walking with the forefoot and to increase energy efficiency and pressure distribution through total contact. The insole was custom-made, and the MIB was 2-mm thick, made of Poron, and placed across the entire width of the insole at the level just proximal to the fourth and fifth metatarsal heads. The gait of the patient was significantly stabilized and nearly normalized immediately after the application of the insoles. The angles of hip flexion were increased, and toe walking and the wide-base gait pattern nearly disappeared (Supplementary Video S1).

## 3. Discussion

In this report, we found disrupted bilateral CRTs in our patient on evaluation using DTT. Furthermore, we presented the successful application of an insole with an MIB and a deep heel cup for treating gait dysfunction in the patient.

DTT (derived from DTI) is a powerful non-invasive imaging technique that visualizes neural tracts in the brain [9,10,11,12]. It is known that all children, even under the age of five years, show well-depicted integrity of the CRT from the cortex to the brain stem level [12]. Therefore, the disrupted bilateral CRTs in our patient seemed to be pathologic lesions. DTT is useful for evaluating a causative brain lesion in pediatric patients with neurologic disorders who have no specific abnormal findings on conventional brain MRI [9,10]. Moreover, pediatric patients under school age frequently fail to undergo physical examination due to poor compliance. In this situation, DTT analysis for evaluating the state of brain neural tracts would be helpful for determining the presence of neurological problems.

Although several studies have demonstrated the disruption of neural tracts in various neurological disorders in children using DTT, the state of neural tracts in patients with DCD or poor coordination has not yet been studied using DTT [13,14,15]. However, some DTI studies have been conducted. In 2012, Zwicker et al. showed that the mean diffusivity of the CST and posterior thalamic radiation was lower in children aged 8–12 years than in typically developing children without DCD [16]. In 2020, Brown-Lum et al. reported that 8- to 12-year-old children with DCD had lower FA and mean diffusivity in the brain areas associated with motor and sensorimotor processing than typically developing children using tract-based spatial statistics [8]. The results of DTI studies have revealed that the microstructural abnormalities in motor function-related neural tracts could be attributed to the development of DCD. Likewise, our patient had disrupted CRTs in both the hemispheres. However, our study differs from previous studies in terms of patient age, which had recruited 8- to 12-year-old patients dignosed with DCD, who were much older than our 23-month-old patient who could not be diagnosed with DCD but had remarkable coordination problems similar to DCD.

In DTT analysis, the target ROI of the CRT is the premotor cortex responsible for coordination, including motor planning. Specifically, the CRT is known to be related to motor functions of the trunk and proximal limbs, such as the hip joint [12,15,17]. Therefore, CRT injury leads to deficits in coordinated motor skills or proximal instability. In 2017, Kwon et al. demonstrated that the CRT is related to trunk stability and gait function [17]. They enrolled 20- to 35-month-old patients with gait dysfunction and age-matched typically developing children. Clinical variables related to trunk stability and gait function were significantly correlated with FA and fiber numbers in the contralateral CRT, but there were no significant correlations with the corresponding parameters of the CST. We believe that the reduced hip flexion during gait in our patient was related to CRT disruption. In addition, trunk instability due to CRT disruption may be the cause of the wide-base gait pattern. We believe that toe walking with strong calf contraction could occur in order to overcome the significant proximal instability that is only present during gait and not observed during standing.

We applied an insole to correct the abnormal gait pattern. We believed that because patients with coordination problems have many limitations in learning and need more repetitive practice than typically developed children, insole application could be an effective therapeutic strategy to automatically repair the corrected gait pattern. In previous studies, custom-made insoles have been shown to assist gait stability by increasing the contact area between the foot and insole surfaces and reducing plantar loading in the foot rather than prefabricated insoles [18,19,20,21]. Our patient showed significantly improved proximal stability and associated hip motion after the application of the custom-made insole. In addition, we used an inverted technique to support supination in the rear foot and pronation in the forefoot so that efficient loading and maintenance could be achieved. He also showed plantar flexion-patterned toe walking and overweight bearing to the forefoot, although he had no definite weakness or spasticity, which is thought to be related to proximal instability from the state of the CRT. Abu-Faraj et al. reported that the metatarsal pad reduced the pressure on the hallux during walking [22]. Manabu et al. reported that abnormal toe walking in stroke patients improved by applying an MIB to an ankle-foot orthosis [23]. In addition, in 2016, Hähni et al. reported that a pad that extends from the medial border to the lateral border of the foot significantly reduces the pressure on the forefoot compared to the conventional metatarsal pad located only in the midfoot [24]. Our elongated MIB could effectively reduce the patient’s forefoot pressure, rather than the conventional metatarsal pad. This reduced forefoot pressure improved the toe-walking pattern and increased the chance of heel contact, which led to a more normalized gait pattern using the ground-reaction force. Moreover, a deep heel cup would enhance the gait stability of the stance phase by positioning the calcaneus firmly in the correct vertical position during heel contact. We think that these applications would eventually have improved the patient’s center of pressure alignment and base of support, which seems to stabilize the gait stability and normalized the gait pattern. In our patient, gait dysfunction showed significant improvement immediately after applying the insole with an MIB and a deep heel cup (Supplementary Video S1).

In addition, treatment using brain-computer interfaces or electroencephalography would be helpful for controlling coordination problems in pediatric patients. These therapeutic tools have the potential to improve gait dysfunction via neurofeedback [25,26].

## 4. Conclusions

In conclusion, using DTT, we found disrupted CRTs and preserved CSTs and MLs in our patient, suggesting that the CRT is related to gait dysfunction characterized by toe walking and decreased hip flexion with a wide base, without definite spasticity or weakness. This gait dysfunction was successfully managed using an insole with an MIB and a deep heel cup. This study is the first to show successful treatment of gait dysfunction in a patient with poor coordination using an insole with an MIB and a deep heel cup. Our study shows the potential of this specialized insole to manage gait dysfunction in pediatric patients. Additionally, we showed that DTT can be a useful tool for determining the causative brain pathology in patients with coordination problems. However, our study has some limitations. First, this was a case report. Second, we did not evaluate improvements in gait dysfunction using gait analysis or other evaluation tool that could explain mote detailed geometric characteristics. Third, the long-term effects of insole-based treatment were not evaluated. Further studies are warranted to compensate for these limitations.

## Data Availability

All data are provided in the paper or in the Supplementary Materials.

## Supplementary Materials

The following are available online at https://www.mdpi.com/article/10.3390/children8050320/s1, Video S1. The patient’s gait before and after the insole application.
